# Foreign

**Published:** 1840-08-22

**Authors:** 


### FOREIGN.

### FERRALL'S CLINICAL LECTURE. No. II.

*Gastritis, subacute, a frequent complication of other diseases—Cases of subacute gastritis treated by depletion and diet—Kreosote in obstinate vomiting—Two forms of organic disease of the pylorus, generally distinguishable during life—Stricture of the pylorus without cancer—Cancer of the pylorus without stricture—Diagnosis.*

Gentlemen,—In the selection of cases for your instruction, I have thought it expedient to bring under your notice what is useful, rather than what is rare, in practice; convinced, as I am, that many pupils quit their studies with their note-books filled with medical rarities, while they are ill prepared by observation for the ordinary duties of their profession. I shall, therefore, occasionally mingle such inquiries with the more serious objects of our researches.

There is an affection of such common occurrence that you can have no pretensions to the rank of a well-informed practitioner without being perfectly familiar with its symptoms and treatment. You may term it subacute gastritis, gastric irritation, or acute dyspepsia, in accordance with different authors; but it is little matter by which denomination you express it, provided you are prepared to recognise its approach, and are competent to its management. It may arise in the course of some surgical disease, or subsequent to an injury. It may complicate a fever, of whatever origin, or (it is in vain to deny it) it may spring up in consequence of the treatment employed to subdue a pneumonia, or some other acute disease. Under any of those circumstances, to overlook gastric disturbance of an urgent character would be to compromise the safety of your patient. You cannot, therefore, bestow too much pains on the study of this important subject.

Three cases of gastric irritation, or subacute gastritis, have been discharged this week, and two post-mortem inspections have been made in cases which were admitted in a hopeless state of organic disease of the stomach. The two latter are instructive specimens of the two varieties of organic lesions of this organ, to which I have before called your attention. You witnessed the difference in their symptoms; you have now the parts before you, and can see whether my explanation of the cause of this difference is founded in reality. I have long been convinced that may cases were termed cancer of the stomach which really had not the essential characters of that malignant disease; and I believe that, in many instances, the phenomena displayed during life were sufficiently distinctive of those affections. The two cases to which I allude have been now under your observation for some time, and you are consequently prepared to follow me in their analysis.

We shall first briefly dispose of the cases of gastric irritation.

*Vomiting—Epigastric tenderness—Loss of flesh—Cure by depletion and low diet.*

Eliza Higginson, æt. 20, was admitted December 5th, complaining of severe pain at the pit of the stomach, loss of appetite, chilliness, and occasional burning heat. She frequently vomits her food, which returns with a remarkably sour flavour. She is thirsty. Her flesh and strength are greatly reduced; skin hot; pulse 90; tongue slightly coated in the centre, with florid tip and edges. The bowels are confined; urine scanty, and high coloured and acid. Epigastrium tender and tumid. She is impressed with the notion that her complaint is all weakness, and has taken ale and porter every day, although she now admits she suffered more in consequence. The catamenia is regular.—She has never been well since she met with an accident by coming in contact with the shaft of a car, about two months ago, but had no other ill consequence.

Cupping on the epigastrium to eight ounces. Arrow-root in small quantities. The bowels to be freed by enemeta, and the following powder to be taken three times daily:

℞. Sodæ Bicarbon. ℈j.; Sacch. Alb. gr. v. M. pulv.

*December* 10th.—She has not had vomiting since her admission. Urine paler, and more copious, but still acid; bowels free; tongue improved in color.

Pergat.

20th.—Feeling herself quite well, she requested a little meat a few days ago, but suffered pain that night; on which account a small blister was applied to the epigastrium. There has been no pain since. The vomiting never occurred since she came into the hospital. Her strength is improving, although her diet is exclusively farinaceous. The skin is dry, but no feverish heat is felt now. Pulse 72. A warm bath is ordered.

24th.—Feels quite well. Discharged.

*Constant vomiting—Epigastric tenderness—Depletion—Kreosote.*

Mary Bardin, ætat. was admitted on account of constant and painful retching, great soreness and pain at the pit of the stomach, and excessive exhaustion. She had been an out-patient for bronchitis. The liquor antimon. tartar, in half-drachm doses, had succeeded in removing the pectoral affection, but the stomach became disordered. She discontinued her attendance at this time, believing that the stomach would improve when she took more liberal diet. This only aggravated the evil; and, after ten days of suffering from vomiting, which gradually increased until even cold water was rejected, she begged to be admitted into the hospital.

The epigastrium was tender; the tongue coated; and, at the tip, studded with red points.—Pulse feeble and small. Skin hot and dry.—Countenance haggard, and expressive of pain. Bowels easily moved, and without pain. Urine scanty.

A sinapism was laid on the epigastrium, and she was directed to take a scruple of aqua lauroceras three times a-day.

The next day the report states that the vomiting is less frequent, but continues still; there is more reaction, and the pulse is more voluminous. She was then cupped on the epigastrium t six ounces, which removed the pain and tenderness altogether. Cold water in sips remained on her stomach, but every thing else was rejected. A drop of kreosote, with mucilage and water, was administered every fourth hour with the best effects; the retching ceased, and arrow-root and boiled bread-and-milk were retained. From this time she improved rapidly. The renal secretion increased under simple treatment, as effervescing mixture, and she was dismissed free from complaint on the eleventh day from her admission.

*Chronic gastritis—Epigastric tenderness—Depletion—Cure.*

Anne Dunne, ætat. 38, had repeatedly suffered from pain in her stomach, with vomiting of sour fluid. On more than one occasion she has attended as an out-patient, and received complete relief from antacids, milk aperients, and counter-irritation of different kinds. Within the last three months she has been losing flesh, and has suffered so much after meat or stimulating diet, that she has given them up altogether. Her countenance is not sallow, though her face is thin. Her tongue is rather clean, but too red at the edge. Pulse 80. Bowels confined. Urine in natural quantity, but depositing deep-coloured lithates. She suffers greatly from flatulence; and has been induced to take stimulants, as peppermint, on account of the immediate relief which follows their use. She has constant thirst, and desire for cold drinks. Catamenia regular. No pectoral disease.

This patient had learned from experience to manage her own diet; and the only error she committed was in the use of stimulants, and in postponing her application for proper aid. She was ordered to remain in bed, was cupped on the epigastrium, and had equal parts of aqua calcis and warm new milk three times a-day. The bowels freed by enemata.

Four days afterwards the report states that the flatulence is very much diminished, and she bears pressure better on the epigastrium. No tumor or circumscribed hardness was to be discovered in that region. The bowels were acted on by emollient enemata, which she found to diffuse a sensation of comfort through the abdomen and stomach.

As the case had now become chronic, and as frequent attacks had succeeded each other for a period of two years, it was deemed advisable to establish a permanent counter-irritation over the epigastric region. For this purpose two moxæ were applied. The pain of this application being of very short duration, is better borne than that from caustics of other kinds.

In a fortnight this poor woman was so far recovered as to desire to return home. The issue was established over the former seat of pain.—Soda was occasionally given in lieu of the aqua calcis, but was always discontinued when the urine showed a neutral or alkaline reaction. Her tongue became pale and moist. She lost the thirst, and was now able to take a little chicken with impunity.

In the latter case, considering the period of life, the frequent recurrence of attacks, and the loss of flesh, there is reason to apprehend that if she relaxes in her attention to diet, or neglects the earliest intimations of irritation, organic disease of the stomach will ensue. We frequently see persons go on for years, occasionally suffering from gastric irritation, and at other times quite free from complaint; until, at length, the attacks settle into disease of an incurable nature. An instance of this kind will occupy us presently, in the case of poor O'Shaughnessy, who died a day or two ago.

The first case, Higginson, showed the advantage of decided treatment, and of depletion whenever the epigastrium is tender permanently.—By this I mean, when the tenderness is not the sequela of an attack of gastrodynia, or spasm, or the soreness which remains after violent vomiting, and which latter often resides in the abdominal muscles. You should not omit depletion by cupping or leeches whenever there is evidence of hyperæmia of the organ, suggested by the florid tongue, thirst, hot skin, local heat over the stomach, and deep-seated tenderness on pressure. This being premised, your other remedies will produce a better effect. The state of the renal secretion will often assist your other diagnosis, although you must not forget that copious fluid vomiting will render this secretion scanty.

The second case, Bardin, evinces the efficacy of kreosote in obstinate vomiting. The advantages of this remedy were first insisted on by Dr. Elliotson, and have since been acknowledged by Dr. Burne, of the Westminster Hospital, who has employed it in irritation both of the stomach and bowels with success.

Of the two forms of organic disease of the pylorus to which I alluded, and which are preceded or accompanied by symptoms of chronic gastritis, one consists in hypertrophy of the tissues entering into this portion of the organ, and appears to destroy the sufferers by increasing constriction of the orifice, impeding the passage of the aliment, and literally starving them to death. The other presents the characters of an essentially malignant change—involves other organs generally—and frequently ends in the dissolution of the patient, without any mechanical obstruction in the part.

*Stricture of the pylorus without cancer—Death from inanition.*

Mary Cousins, æt. 39, stated on admission that she had been labouring for six months under her present complaints. They commenced by a sudden attack of vomiting without any evident cause. She had pain in the stomach, but does not describe it as having been severe. This acute attack gradually subsided without treatment, but left a disinclination for food, and pain after eating. The vomiting, which during the acute invasion brought up everything, solid and fluid, immediately it was swallowed, was then much less frequent, and only followed the ingestion of animal or stimulating food, and she began to think herself well. But latterly, milder food returns, and she suffers pain after every meal. She is remarkably emaciated, and describes the change in this respect as being very great within the last month; her features are drawn and lengthened; the nose is blue, as if from cold; the lips pale; tongue slightly coated, with red edges; pulse small, 100; skin dry; bowels obstinately constipated: she says three weeks elapse between the evacuations. On examination, a hard tumor, about the size of a small apple, is found to the right of the umbilicus; there is tympanitic distension, especially of the left side of the abdomen; very little pain on pressure.

This was the report of her state on admission. Several days elapsed before the bowels yielded to enemeta, and then a very large fæculent discharge took place; a dose of aqua lauro-ceras, before her slight meal of arrow-root, seemed to cause it to be retained, but we found on inquiry that the vomiting was only postponed. She did not vomit after every meal as heretofore, but it gave her pain; and now an attack of vomiting was observed to occur regularly at 5 o'clock every morning. On inspection, the quantity ejected was found to exceed greatly what had been ingested; it consisted of a brown fluid, like chocolate, mixed with mucus and some traces of the food; the bowels remained confined, and acted not more than once a week; the urine brown and scanty; the tongue generally dry. The saliva sometimes yielded an acid reaction, but nothing satisfactory could be said on this point, from the variable nature of the results. She suffered from thirst, but dreaded swallowing on account of a peculiar distress which followed it: together with the pain, a remarkable sensation of regurgitation immediately commenced (and could be felt by the hand laid on the abdomen) at the seat of the hard tumor; from this point it ascended towards the cardiac orifice, and thence returned again. Nothing could exceed the dread she felt of exciting this painful state.

I need not remind you of the various remedies employed to soothe her sufferings. Preparations of hydrocyanic acid, bismuth, kreosote with opium, belladonna externally and internally, &c. &c.; all of these produced relief for a time, but I think she derived more frequent comfort from kino and acetate of morphia. This copious secretion of the mucus, as well as the flatulent distension of the stomach, appeared to be in some measure diminished by this plan. The chocolate-coloured vomiting, however, persisted, and was only occasionally controlled by the exhibition of the tincture of the muriate of iron.

With a view to sustain this poor woman, enemata containing strong broths, milk, and arrow-root, were tried, and seemed for a while to revive her; at length she began to sink more decidedly, her voice became husky and reduced to a whisper; her pulse scarcely palpable; her tongue icy cold; the surface, generally, cold and bluish, and reminding us of the collapse of cholera; the evacuations by the bowels more rare; occasional hiccup. She lay for several days in a torpid state, sleeping with the lids half closed, and scarcely living. The vomiting occurred regularly until she was released from her sufferings.

*Autopsy.*—Remarkable congestion in the veins of all the intestines as well as of the stomach. The vessels large, and marked by a brownish dark blood. The stomach was enormously distended, and taking an unusual course. The whole left half of the abdomen was occupied by this organ, which descended directly into the left iliac fossa; thence it ascended towards the umbilicus, to the right of which was situated the hard tumor of the pylorus, which had been felt during life. The stomach contained a large quantity of brown fluid similar to what has been ejected; the mucous coat was tinged of the same colour; the veins large and congested.—The thickness of the stomach in its pyloric third was such as to make it resemble leather to the touch. A section of this portion, you perceive, reminds us of a thickened urinary bladder with deceased prostate gland; the passage into the duodenum would hardly allow the passage of a probe; the contracted portion was near three-quarters of an inch long, and ascended by a curve to the duodenum.

On examining the section carefully, you perceive the tunics are all thickened, but there is no breach of surface. The mucous coat presents no trace even of abrasion. The brown chocolate fluid was then an exhalation from the surface of the membrane. You cannot dissect off the mucous membrane without the sub-mucous cellular coat. They came off together, and appear to be connected with the muscular coat by a fine cellular tissue. This is not exactly what Andral describes; for he mentions the thickened cellular tunic dipping by bands between the muscular fibres, and connecting itself with the sub-serous cellular tissue. The muscular coat, when washed, presents very much the colour and appearance of the muscle of a fish; the fibres are distinct, but hypertrophied. The thickness of the section is rather more than half an inch at the pylorus, and rather less than half an inch below that point.

The liver was dark coloured, but contained no morbid deposit. The gall bladder was very large, and filled with deep-yellow-coloured bile; the spleen of moderate size; the kidneys rather small, and of a venous colour on section, but presented no other change; pancreas healthy.

We shall now pass to the case of O'Shaughnessy, and conclude by contrasting it with the former.

*Cancer of the pylorus without stricture.*

Ellen O'Shaughnessy, æt. 40, was a patient in St. Vincent's Hospital on a former occasion, (twelve months ago). She suffered, at that time, from pain after food, tenderness below the ensiform cartilage, distressing burning sensation, and acidity. This state succeeded to a rather sudden attack of pain and vomiting, which she attributed to interruption of the menses. She never ventured to use animal food afterwards without suffering immediate pain and burning heat, and, within half an hour, rejecting it entire. She applied to a medical gentleman for advice in the beginning, but states that no depletion was employed. On her admission for the first time into the hospital, she had leeches applied, and blisters, and took small doses of opium, with antacids, with good effect. Her appearance improved, though not very much, and she returned home, feeling quite well, but exhibiting a sallowness of complexion which did not belong to health; farinaceous diet was advised.

She then went to attend a lady, who pressed her to use a more nourishing diet. In deference to this advice she tried wild fowl in small quantity, but soon wished to give it up, on account of returning pain. Her patroness insisted on her persevering, and assured her of success, alleging that she only wanted proper nourishment to restore her completely. The pain, however, increased; she became feverish again, lost colour and flesh rapidly, and, although the vomiting seldom occurred now, her suffering was so great, after even the mildest food, that she lost rest altogether.

In this state, after an absence of twelve months, she begged to be re-admitted into the hospital. She was now so altered in appearance, that she was not recognised at first. Her voice was feeble; her colour sallow. She was emaciated, and her countenance wore the aspect of malignant disease.

The abdomen, on examination, afforded abundant evidence of organic disease; a large tumor occupied the epigastric region, and could be traced upwards under cover of the ensiform cartilage and ribs. The whole of this region was dull on percussion, as well as the right hypochondrium; a distinct bulging prominence marked the centre of this solid mass. The abdomen was rather shrunken, and had little of a tympanitic sound. The pain she complained of extended, she said, through the bowels towards the pubic region. She had occasional diarrhœa and griping, but not constipation at any time. It was evident that extensive organic disease existed in the abdomen; the bulging prominence was considered to be a tuber of the liver: the situation of the pylorus could not be ascertained.

The principal indications, namely, to moderate the diarrhœa. and lessen the sensibility of the mucous surfaces, were attempted by cretaceous preparations, with opiates, aqua calcis, rice, flour and milk, &c., with varying success. Every remedy seemed of use at first, but soon ceased to exert any control over the symptoms. The same remedy was often resorted to again with advantage, although it had, before, ceased to procure relief. The diarrhœa was remarked to produce very fœtid discharges. The renal secretion was generally scanty, and remarkably loaded with purpurates; her colour now became more jaundiced; she had occasional perspirations at night, and lost strength rapidly; she had hiccup. The pain was absent occasionally for several days together, and certainly became much less distressing. For two or three weeks before her death she had no vomiting; but the diarrhœa became less manageable even by opiate enemeta. The dejections became paler, but still very fœtid. The tongue dry and brown; the pulse faltering; the hiccup more frequent. She sank in a semi-comatose state. The saliva was occasionally acid during this period.

*Autopsy.—Head.*—The vessels of the brain (the veins and sinuses) were rather turgid; no remarkable arterial vascularity. The consistence of the brain was rather firm; no sub-arachnoid or other effusion.

*Chest.*—The pleuræ costalis and pulmonalis were adherent at several points of right chest; a few tubercles were found in a crude state in the apex of that lung. No disease in the left side. The heart was rather small; muscular substance softer than natural; cavities and orifices normal.

*Abdomen.*—The peritoneum was adherent in a few places, and connected the liver to the diaphragm. The liver was large, and presented a striking example of the soft tubercle. You observe the masses are circumscribed accurately, have a yellow colour, are depressed in the centre, and are marked by numerous radii formed of blood-vessels proceeding from the liver towards the centre of the morbid growth.—Their circumscription, in the substance of the liver, is complete; for you perceive they can be turned out entire, and without destroying anything but a very fine cellular tissue which surrounds them as they lie embedded in the organ; a section exhibits a yellow soft encephaloid substance, almost creamy in some parts, enclosed by septa of a firmer consistence and a whiter hue. There is no very apparent arrangement of the septa. Some of the tubera are excavated in the centre, or softened into a puriform fluid. The stomach was rather contracted; it lay concealed by the liver, and by a mass of morbid growth which surrounded the pylorus, and extended behind it towards the spine. Several globular tubera, as large as filberts, were found in this mass: their structure was similar to those in the liver. The gall-bladder was small, and contained a little dark green bile. On opening the stomach, a large sloughy excavation occupied the place of the pylorus; the surface was irregular; in some places reduced to a pulp; and in others, where sloughing had not occurred, gristly to the feel. This large ulcerous cavity was smeared with a peculiarly fœtid sanies. The passage of the pylorus was open and large, and admitted two fingers to pass readily into the duodenum. A section of this mass showed a surface of cartilaginous appearance, striated, but exhibiting no trace of the tunics of the organ. The stomach exhibited little thickening beyond the seat of this cancerous disease. The mucous coat of the intestines was tumid and vascular, towards the termination of the ileum; no abrasion was discovered. The spleen and kidneys presented nothing remarkable.

You will now, gentlemen, understand why I desired your special attention to the phenomena which, so very different from each other, characterize those cases during life. You will also be convinced of the value of minute anatomical analysis, post-mortem, by the clear and satisfactory explanation which, in these two cases, it affords of the symptoms you observed.

In the case of Cousins, the pylorus, though less extensively diseased, could be felt during life, because, in the first place, no other tumors existed to conceal it; and secondly, because the enormous distension of the stomach, occasioned by the permanent obstruction at its orifice, brought it lower down into the abdomen. But how are you to recognise a pyloric tumor, so far displayed inferiorly, as to be on a plane with the umbilicus, and latterly, as to be placed in the right side of the middle line? Might it not be a tumor of the omentum, or other growth connected with the intestines? If you adopt this rule, you will seldom be deceived. Percuss the proper region of the stomach, and discover its peculiar tympanitic clear sound. Then follow this sound, by progressive percussion downwards, till you lose it, and finally pass to the right, till your fingers are conducted by this sound to the spot occupied by the pyloric tumor.

It is to Andral we are indebted for precise notions of hypertrophy of the tunics of hollow organs, as distinguished from other changes of a more malignant nature. He remarks the enormous distension of the stomach, which occurs in pyloric obstruction from this cause, and records examples where the contents were rejected only every seven or eight days. He also notices the fact, that chocolate or coffee-coloured fluids may be vomited in cases which present no abrasion of the mucous surface.—The case of Cousins is well calculated to support his opinion, and shows that this kind of vomiting is not, as it was considered in Dr. Baille's time, pathognomonic of cancer of the stomach. Dr. Baillie does not appear to have recognised this disease. He describes stricture of the pylorus as caused by "permanent contraction of its muscular fibres," and accounts for its occurrence at the pylorus, more frequently than at the cardia, by the "fibres at the pyloric end being more circular in their direction," and possessing "a stronger contractile power." He adds, "I have seen one instance of this contraction at the pylorus, which, even there, is a very rare disease." It is difficult to say whether this is meant to apply to a case of hypertrophy, or whether he may not have been deceived by the contracted state of the pylorus, which often exists at the moment of death; but which did not exist, perhaps, an hour before that period, and which is easily removed after death, by the slightest dilatation, without injury to the parts. I believe this to be possible, because

I have more than once seen a contracted state of this orifice exhibited as a disease, when the largest finger could be readily passed through it, and when the tunics, thus separated, were perfectly natural in thickness, pliability, and consistence. In the section entitled, "diseases of the pylorus," that distinguished pathologist, Dr. Ambercrombie, describes some of the prominent symptoms; but does not contemplate any distinction between the cases in which hypertrophy without cancer is to be found, and those in which the essential characters are malignant. Indeed he does not appear to have examined the parts with that minuteness which alone could have enabled him to make the distinction. He gives three cases, and in the first states merely that "the pylorus was surrounded by a mass of schirrus, the size of an orange, very firm, or nearly cartilaginous;" "the stomach in other respects was entirely healthy." In the next case, he says, "a mass of schirrus, four or five inches in diameter, surrounded the pylorus, and the pyloric orifice was so narrowed as scarcely to admit the point of a very small finger. The inner part of the mass opened upon the internal surface of the stomach by an ulcerated space, covered with large cancerous-looking tubercles. The other parts of the stomach were tolerably sound."—In the third case also, he describes the external tumor and the internal surface, or that presenting into the cavity of the stomach; but the structure of the mass is not displayed by a section, by which, and a short maceration, the true nature of the tumor can alone be determined.

Returning then to the two cases before you, you will observe that they differ from each other in anatomical characters, in localization, in their progressive changes, in the cause of death, and in certain symptoms manifested during life.

In the first case (Cousins) there is hypertrophy of the tissues, but they are distinguishable: it is as if they were placed under a lens and enlarged. In the second case the tissues are disorganised, and confounded together.

In the first case, the disease is local, and seems to rise from gastritis of long duration. In the second case, the taint is general, and other organs are involved in the malignant change.

In the first case, the disease ran its course to a fatal issue, without any breach of the mucous surface. In the second case, ulceration was probably an early change, for the opening was considerably enlarged.

In the first case, the patient died from irritation, the pyloric orifice being almost completely closed. In the second, the passage was larger than natural, and the patient sank under the disease of several organs, and the general taint.

In the first case, the patient had a peculiar, cold, pinched, and starved look; the vomiting continued up to the time of death, and the whole ingesta were returned; and there was constipation, or, I should prefer calling it, rarity of fæcal discharges, for constipation means a different state. In the second case, the aspect of the patient was that of malignant disease; the vomiting became less frequent, and ceased before death; and there was an opposite condition of the bowels, or diarrhœa*.

I may remark that, in cases like the first, the vomiting of each meal separately may be generally prevented by suitable remedies; but you must not be deceived by this event; for the food will no less certainly return, as soon as the stomach becomes so much distended as to bear no further accumulation; and this I have remarked to occur more especially in the horizontal posture.

The state of the gall-bladder is worth a word or two of notice. It was found in the first case greatly enlarged and distended with bile. Now this appears to be connected with absence of chyme in the duodenum, and the want of the accustomed stimulants to the biliary ducts. Morgagni relates the experiment performed by Valsalva on a dog which he, cruelly enough, starved to death. The gall-bladder was unusually large, and distended with bile. The starvation in poor Cousins's case was the result of organic obstruction. The celebrated surgeon Mr. Carmichael, suggests as an explanation of the occurrence of gall-stone in the human subject, the pernicious habit of long fasting and late dinners, which so generally prevails. I have no doubt that, in many instances, gall-stones have their origin in this way; but some other cause must occasionally coincide to produce the result. In Cousins's case, for instance, there was long and painful fasting, and the gall-bladder was found distended with bile, but there was no gall-stone in that case.—*Lon. Med. Gaz.*

---

*Case of Extirpation of the Uterus.* By M. Luytgaerens of Zèle.—Josephine Vans Mousselde, of a good constitution, and about 36 years of age, was, after a laborious labour, delivered of her first child in the year 1835. She enjoyed good health till about the end of the year 1837, when she began to suffer from uterine discharges, consisting of blood, or blood mixed with purulent matter, accompanied by pains and a dragging sensation caused by a tumour in the vagina.

In the month of August, 1838, she applied to a physician, who recognised the presence of a

* Subsequent to the delivery of this lecture, a series of preparations and drawings, and cases illustrative of these two pathological states, were laid before the Pathological Society of Dublin, (see Dub. Journal,) and later still I have had the satisfaction to observe that Dr. Addison, the distinguished Physician of Guy's Hospital, has arrived at similar results, in arranging the two classes of disease.

large fleshy polypus attached to the fundus of the uterus, and which, by its weight, had caused a complete prolapsus of that organ. The tumour projected externally, more or less, according to the position in which the patient was placed. It was about the size of the fist; largest below, and contracted above where it adhered to the uterus. At its upper end a hard circular ring was felt, apparently of the *os uteri*, between which and the tumor the finger could be pushed. From this circumstance it was believed that the uterus itself was reversed, and constituted the upper portion of the tumour. All the parts were covered with an abundant sanies, exhaling a fœtid gangrenous odour; hectic fever had already come on, and the patient was evidently sinking under the disease. Extirpation of the tumour was therefore resolved on as the only means of saving the life of the patient, and the operation was performed in the following manner.

The patient was laid on her back on the edge of the bed, the thighs well separated, and the tumour seized with a double forceps of Museux; the whole mass was then drawn beyond the external organs of generation, and, being held fixed in this position, was extirpated by means of a strong pair of curved scissors.—Scarcely was the first cut made, when a cavity was opened into; a circumstance which showed that the uterus was included in the section. A finger was introduced into the cavity by the incision, and directed upwards, but it was found that adhesion had taken place, which prevented it reaching the common peritoneal cavity. The operation was then finished, ligatures being applied to each artery as it was cut through.

The operation was not followed by hæmorrhage nor any other unfavourable event. The pain was easily allayed by opiate and emollient injections, and by opiates administered internally, and in ten days the cure was complete.

The third month after the operation the menstrual flux reappeared but in very small quantity; and six months after the operation she was in the enjoyment of good health; her menstruation was regular, but in very small quantity; and she was able to follow her ordinary occupation.—*Edinburgh Med. and Sur. Jour. from Annales de la Societè de Medicine de Gand.*

---

*On the influence of the Manufactories on the developement of Children.* By M. Dulpin.—The pernicious effects which result from confining children in manufactories, both as influencing their health and growth, have been pointed out by M. Dulpin in his interesting work on this subject. One fact alone may be stated here, to show the value of statistical researches in elucidating the subject.

M. Dulpin chose ten agricultural districts and ten manufacturing districts, and in these ascertained with great care the number of men of 20 years of age who were rejected as recruits on account of deformity or general unfitness for military service. In the agricultural districts, in order to furnish a complement of 10,000 recruits fit for military service, it was found that 4029 were rejected as infirm or deformed; whilst in the manufacturing districts, to furnish the same number of men, it was necessary to reject 9930 as infirm or deformed.

In some of the departments, however, the number of men infirm or deformed was much greater. Thus to obtain 10,000 men capable of carrying arms in the department of the Marne, 10,309 were rejected as infirm or deformed; in that of the Lower Seine, 11,990; and in that of the Eure, 14,451.

In the towns of the department of the Lower Seine, the number rejected rose much higher. Thus, to obtain 100 men sufficiently strong to carry arms, it was necessary to reject, on account of weakness or deformity, at Rouen, 170 young men of 20 years; at Elbeuf, 200; and at Belbec 500.—*Ibid.*, *from Comptes Rendus*, 13th April, 1840.

---

*Calomel with Iodine and Sugar.*—This combination is employed in Riga, for the hydrocephalus of children, with marked success. The usual description is as follows:—

℞. Calomel, gr. viij., Iodin. gr. j., Sacchari albi, gr. lxxx. M. ft. pulv. divid. in xvj. partes æquales.

Powdered digitalis with *pulvis gummosis* are sometimes combined with it. If the calomel is first rubbed with the iodine, and the sugar then added, the powder becomes red; but if the calomel is first mixed with the sugar, and then the iodine added, the colour is greenish; deuto-ioduret of mercury being formed in the first case, and the proto-ioduret in the second. This suppositon has been pretty well confirmed by analysis. The red powder has effected the greatest number of cures. According to theory, eight grains of calomel, mixed with one of iodine, should afford, of

Protochloride of mercury (calomel,) 6.124 gr.
Deutochloride of mercury (corrosive sublimate,) 1.078 gr.
Deuto-ioduret of mercury, 1.798 gr.

The only difference in the result of actual analysis was, that the powder contained a trace of proto-ioduret of mercury, and in consequence rather more corrosive sublimate than the theory supposes.

As each powder is made, according to the above description, with half a grain of calomel and one-sixteenth of a grain of iodine, it contains, after the manipulation,

Corrosive sublimate 0.067 or about 1-16th of a grain.
Calomel 0.383 or about 2-5ths of a grain.
Red ioduret of mercury 0.112* or about 1l9th of a grain.

*Lon. Med. Gaz. from Schmidt's Jahrbücher.*

* By an error of the pen or the press, this is 383 in the original.—Translator.

*Disease of the Kidney simulating Lumbar Abscess.*—At the Pathological Meeting of the Medical and Chirurgical Society, June 16th, Mr. *Benjamin Phillips* presented a preparation of the kidney illustrating the following case:—The patient, a man, 55 years of age, had been for many years a horse patrol; during the previous two or three years he had suffered occasionally from pain in the small of the back, which was suspected, by the surgeon in attendance, to result from disease in the kidneys; he was cupped two or three times with slight relief. When admitted into the Marylebone Infirmary the pain was still troublesome, there was little tumefaction or fluctuation, and the urine presented no remarkable change. When he had remained in the institution some weeks the disease assumed a more definite character, the pain in the loins became more severe, tumefaction and fluctuation became apparent, and lumbar abscess was suspected. It now became a question whether the tumor should be punctured, or allowed to burst; it was determined that a small puncture should be made, when a small quantity of thin, offensive, purulent fluid escaped; the puncture closed, and no constitutional irritation resulted. After some weeks a collection of fluid again took place, and made its way over the entire of the back, from the sacrum to the top of the scapula. The tumor was again punctured, and a pint and a half of the same kind of fluid, as before, removed; the puncture closed, no constitutional irritation followed, but at the expiration of ten or eleven days, there was found to be ulceration at the point where the opening had been made, from which there was a constant dripping of fluid, amounting sometimes to as much as a pint during the day. He went on in this way for nine or ten weeks, hectic coming on only during the last fourteen or fifteen days; during the whole of this period there was no remarkable change in the urine. He died from the irritation induced by the disease, which was still thought to be lumbar abscess with disease of the vertebræ.

After death, the abdomen was first laid open, and a long probe introduced into the fistulous opening in the back, just above the sacrum; the probe passed directly into the substance of the kidney, on examining which it was found to contain three or four large calculi; one of these was close to the surface of the left kidney, and had produced irritation, inflammation, and consequent suppuration in the neighbouring tissues. There was slight disease of the lumbar vertebræ near the kidney.

The case was interesting from its illustration of the difficulty of determining, during life, whether the abscess was dependent upon disease of the vertebræ or of the kidneys. The fact of the urine remaining healthy was a strong presumption in favour of the kidneys being unaffected. He was not aware of any means of diagnosis by which, in such cases, we could distinguish the exact seat of the mischief.

Dr. *Clendinning* had a curious case of disease of the kidney under his care two years since, in which he was able to determine the nature of the malady. The patient, a man, complained at first only of debility; pain in the renal region then came on, and a tumour appeared in that part. Swelling became dull on percussion, and when emaciation had proceeded to a great extent, the kidney could be seen quite plainly, very much enlarged, through the integuments of the abdomen. After death the kidney was found much increased in size, and its pelvis was occupied by large calculi, which, from their irritation, had produced inflammation and suppuration of the organ.

Mr. *Gregory Smith* had seen three cases of abscess in the kidney resulting from the presence of calculi in the organ. One of these cases was that of a patient who was admitted, some years since, in a state of hectic, into St. George's Hospital. There was a large abscess pointing, and apparently about to burst in the lumbar region. Sir B. Brodie laid the abscess freely open, and on introducing his finger into the cavity, detected several loose bodies at the bottom of it; these being removed by means of a small forceps, proved to be three calculi of the size of nutmegs. The patient, a woman, lingered a short time, and sunk. The structure of the left kidney was found to be destroyed by suppuration, and there was no communication with the bladder, the ureter being impervious. In Mr. Phillip's case part of the kidney was still so far normal in structure, that it would probably perform its function. Was there any trace of purulent deposit in the urine in this case? In another case he (Mr. Smith,) recollected, in St. George's Hospital, the kidney had become enlarged to the size of a two-quart bottle, and contained a calculus which had moulded itself to the interior of the kidney. Cases of abscess pointing in the loins were interesting from the fact of their liability to be mistaken for disease of the vertebral column. He thought, however, the symptoms in the first class of cases were more of a typhoid character than they were in the second, and this would assist in our diagnosis.

Mr. *Arnott* remarked, that the case detailed by Mr. Phillips, and those of a similar character, were interesting chiefly from the difficulty which the practitioner experienced in determining the origin of the disease during the life time of the patient. He thought that where the abscess depended upon disease in the kidney, it was much more rapid in its progress than when it was the result of disease of the vertebræ.

Mr. *Phillips* said, there would still be a difficulty in this mode of diagnosis, from ignorance of the time at which we should commence our observation of the rapidity of the suppurative process.—*London Lancet.*

